# Acquired cystic kidney disease in children with kidney failure

**DOI:** 10.1007/s00467-024-06628-7

**Published:** 2025-01-04

**Authors:** Justin Ming-yin Ma, Kin-fen Kevin Fung, Pak-Chiu Tong, Wai-ming Lai, Alison Lap-tak Ma, Eugene Yu-hin Chan

**Affiliations:** 1 Paediatric Nephrology Centre, Hong Kong Children’s Hospital, Hong Kong, Hong Kong SAR; 2 Department of Radiology, Hong Kong Children’s Hospital, Hong Kong, Hong Kong SAR; 3 Department of Paediatrics, The Chinese University of Hong Kong, Shatin, Hong Kong SAR

**Keywords:** Kidney failure, Children, Acquired cystic kidney disease, Renal cell carcinoma, Dialysis

## Abstract

**Background:**

This study aimed to evaluate the incidence, contributing factors, and clinical outcomes of acquired cystic kidney disease (ACKD) in children undergoing kidney replacement therapy (KRT).

**Methods:**

We conducted a cross-sectional, territory-wide study at the designated pediatric nephrology center in Hong Kong. ACKD was defined as the presence of ≥ 3 cysts in the native kidneys, excluding congenital or hereditary cystic diseases. Between June to December 2023, all paediatric patients receiving KRT in Hong Kong underwent ultrasonography, non-contrast magnetic resonance imaging (MRI), or both. Contrast-enhanced computed tomography was performed for patients with complex cysts.

**Results:**

Forty-three children (56% female; median age 14.7 years; IQR, 11.7–18.7) were included in the analysis. ACKD was detected in 18 children (42%). Nine subjects had complex cysts (grade 2, *n* = 5; grade 2F, *n* = 2; grade 3, *n* = 2). Most patients with ACKD (89%) were asymptomatic. One patient (5.5%) developed back pain and gross haematuria 72 months after initiation of KRT. Another patient (5.5%) developed infected cyst with back pain and clinical sepsis 60 months following KRT initiation. A dialysis duration of ≥ 28 months was the only significant factor associated with ACKD development (77.8% vs. 40%; *p* = 0.028; OR_adj_ 6.09, 95% CI 1.43–25.82, *p* = 0.014). The diagnostic yield of paired ultrasound and MRI was superior to ultrasound alone.

**Conclusions:**

ACKD is prevalent among children and adolescents with kidney failure, with most cases being asymptomatic, however serious complications may arise. Longer duration of dialysis is significantly associated with ACKD development. Therefore, early transplantation and active ACKD surveillance are crucial for children receiving KRT.

**Graphical abstract:**

**Figure:**
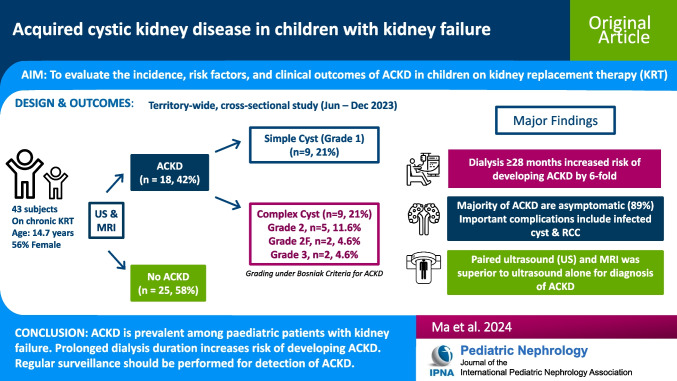
A higher resolution version of the Graphical abstract is available as Supplementary information

**Supplementary Information:**

The online version contains supplementary material available at 10.1007/s00467-024-06628-7.

## Introduction

Cystic kidney disease in childhood is a heterogeneous group of conditions and can be hereditary, congenital (non-hereditary) or, less commonly, acquired in nature [1]. Acquired cystic kidney disease (ACKD) is defined by the presence of multiple small cysts in bilateral small kidneys in patients with kidney failure who do not have other hereditary or congenital cystic disease [2]. It is a serious complication of kidney failure, which is a rare condition in childhood associated with mortality and significant morbidities such as infection and cardiovascular disease [3–9]. The previously reported incidence of ACKD in children ranges from 21.6 to 45.8% [10–13], while its incidence can be up to 90% among the adult dialysis population [14].

The underlying pathogenesis of ACKD remains unclear, and uraemia has been hypothesized to be the key mediator for cyst formation [15, 16]. Nephron loss of any cause may lead to compensatory hypertrophy in the remaining normal nephrons [17]. This process is regulated by growth factors and proto-oncogenes, which may lead to tubular hyperplasia and cyst formation over time [18]. The risk of developing ACKD significantly increases with the duration of maintenance dialysis [17, 19–21]. ACKD can lead to serious complications such as infection, cystic rupture, cystic haemorrhage, perinephric hematoma, and renal cell carcinoma [22]. Despite the gravity of this condition, data pertaining to ACKD in children are scarce. There is also a lack of consensus on the surveillance protocol in paediatric ACKD.

This aim of this study was to investigate the incidence, associated factors, and clinical outcomes of ACKD in children with kidney failure.

## Methods

We conducted a territory-wide, cross-sectional study at the Paediatric Nephrology Centre of Hong Kong Children’s Hospital, Hong Kong SAR. The Paediatric Nephrology Centre, previously located in the Princess Margaret Hospital, is the sole referral centre for paediatric maintenance kidney replacement therapy (KRT), namely peritoneal dialysis, haemodialysis, and kidney transplant in Hong Kong. Our study included all children and adolescents aged less than 18 years at the time of KRT initiation, who were actively being followed during the study period between June 1st 2023 to December 31st 2023. Patients with hereditary cystic kidney disease (such as nephronophthisis, autosomal dominant cystic kidney disease, or autosomal recessive polycystic kidney disease), congenital anomalies of the kidney and urinary tract (CAKUT) with pre-existing cystic changes, bilateral nephrectomies, or those who were lost to follow-up, were excluded. Eligible patients were identified by the existing database. We classified patients according to their primary kidney disease. In patients with CAKUT who had an underlying genetic variant identified, the cases were classified as CAKUT. The study was performed in accordance with the Declaration of Helsinki and was approved by the Institution Review Board of the Hong Kong Children’s Hospital, Hospital Authority, Hong Kong SAR (Reference no. HKCH-REC-2020–011). Written consents were obtained from all study participants, or their guardians, as appropriate.

We offered both ultrasonography and non-contrast magnetic resonance imaging (MRI) of native kidneys for all recruited patients. Patients were consented to undergo either or both imaging modalities. ACKD was defined by the presence of three or more cysts in each of the bilateral native kidneys, among patients with kidney failure without underlying cystic disease [23]. Among patients with ACKD, the cysts were further categorized as simple or complex cysts based on imaging appearance on ultrasonography or MRI, following the Bosniak classification version 2019 [24] (Table 1). In case of cysts of grade 2 or above, contrast-enhanced computed tomography (CT) was performed to delineate the cyst structure and allow accurate grading assignment. Final assignment of Bosniak grading was based on the highest grading of all evaluated cysts. Patients who had no cyst or less than three cysts (solitary cysts) in each kidney were classified as not having ACKD.

**Table 1:** The Bosniak kidney cyst classification [15, 24]

Grading	Features	Malignancy Risk
**Simple**		
I	Well defined, thin (< 2 mm) smooth wall Homogenous simple fluid No septa or calcifications	< 1%
**Complex**		
II	+ Cystic masses with few septa (1–3) Hairline septa (< 1 mm) Fine calcifications	< 3%
IIF	Smooth minimally thickened (3 mm) enhancing wall or septa; increasing number of septa; thick calcifications	5–10%
III	1 or more enhancing thick walls septa (> 4 mm) or enhancing, nodular, irregular septa	40–60%
IV	Clearly malignant: solid mass with a large cystic or a necrotic component	> 80%

The primary outcome was the incidence and grading of ACKD, while the secondary outcomes included clinical presentations, complications, and association factors such as the duration of chronic dialysis. Anonymized data concerning patient demographics, clinical manifestations, and laboratory results were obtained from the electronic medical record system (Clinical Management System of Hospital Authority in Hong Kong).

### Statistical analysis

Statistical analysis was performed by IBM SPSS Statistics version 27 software. Statistical significance was considered for *p*-values of less than 0.05. Patients were categorized as having simple cysts, complex cysts, and non-ACKD. The descriptive statistics for this study included frequencies (percentage) for categorical variables and median (interquartile range, IQR) for current age, age at KRT initiation, and duration of dialysis until the last follow-up. To assess the unadjusted association of each independent variable for patients with ACKD and patients without ACKD separately, univariate tests were initially conducted. Fisher’s exact tests were used for the analysis of categorical variables, while Mann–Whitney U tests were used for comparing current age, age at KRT initiation, and duration of dialysis until the last follow-up. For subgroup analysis of comparing simple cysts, complex cysts, and non-ACKD, the Freeman–Halton extension of the Fisher exact probability test for categorical variables and the Kruskal–Wallis test for continuous variables were used.

Univariate analyses were performed to evaluate the association between patient characteristics and the diagnosed ACKD outcome, with odds ratios calculated for each characteristic. To account for the small sample size and estimate adjusted odds ratios (AOR) with a 95% confidence interval (95% CI), multivariable analyses were conducted using a multiple logistic regression model. The variables included in the model were gender, age, duration of dialysis, and history of kidney transplant.

Due to the possibility of odds ratios overestimating the risk, as the incidence of the outcome increases, multiple logistic regression models were employed to estimate adjusted relative and attributable risks for the outcome. The optimal cut-off for the duration of dialysis for ACKD patients was evaluated by the receiver operating characteristic (ROC) curve (Supplementary Fig. 1).

## Results

### Patient population

**Fig. 1:**
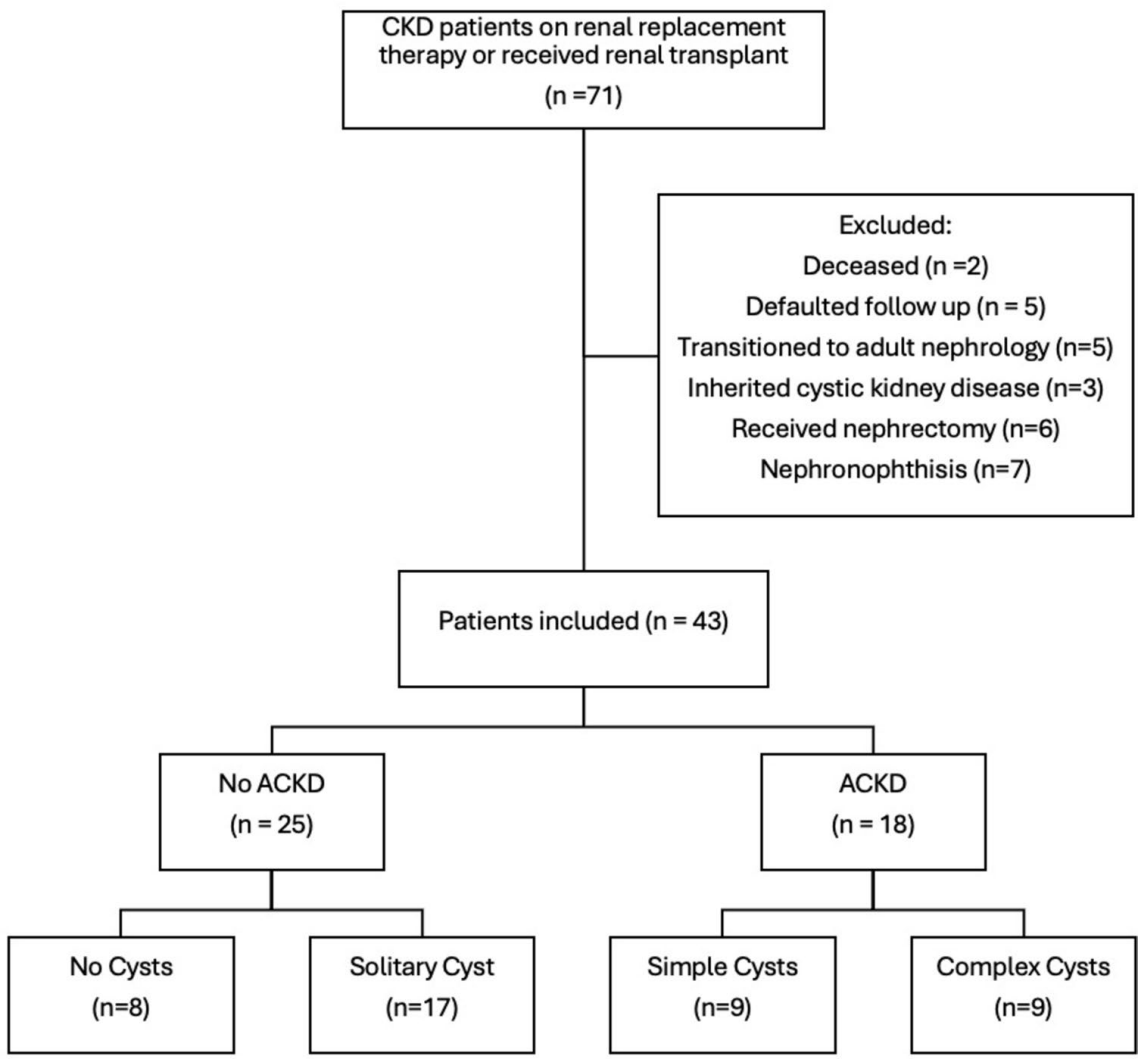
Inclusion and exclusion flow chart

A total of 71 children and adolescents with kidney failure were identified. Forty-three patients were included in the final analysis (Fig. 1). Twenty-four patients (52%) were female, and the median age was 14.7 years (IQR, 11.7–18.7) at the time of evaluation. The median age for KRT initiation was 8.7 years (IQR, 4.5–14.3). The majority of the patients were Chinese (88%). In terms of primary kidney disease, 15 patients had CAKUT without cystic changes in the kidneys at baseline (34.9%), 11 patients had glomerular disease (25.5%), 4 patients had hereditary nephropathy (9.3%), and 13 patients had miscellaneous kidney diagnoses (30.2%). Twenty-five patients (58.2%) were kidney transplant recipients. The median duration of dialysis at the time of evaluation was 29 months (IQR, 17.0–46.5). Before the initiation of KRT, solitary kidney cyst(s) were detected in six patients. Of these patients, 3 subjects further progressed and fulfilled the diagnosis of ACKD after KRT initiation (Table 2).

**Table 2:** Baseline demographics

Patient characteristics (*n* = 43)	Overall (*N* = 43)		ACKD (*N* = 18)		Non-ACKD (*N* = 25)		*p*-value
Gender: Male, *n* (%)	19	(44.2%)	7	(38.9%)	12	(48.0%)	0.76
Age at 31 Dec 2023 (year), median, (IQR: Q1–Q3)	14.7	(11.7–18.7)	14.3	(10.0–19.8)	14.7	(12.5–18.4)	0.92
Ethnicity, *n* (%)							0.63
Chinese	38	(88.4%)	15	(83.3%)	23	(92.0%)	
Non-Chinese	5	(11.6%)	3	(16.7%)	2	(8.0%)	
Caucasian	1	(2.3%)	1	(5.6%)	0	(0.0%)	
Nepal	1	(2.3%)	0	(0.0%)	1	(4.0%)	
Pakistani	3	(7.0%)	2	(11.1%)	1	(4.0%)	
Primary disease, *n* (%)							
CAKUT	15	(34.9%)	7	(38.9%)	8	(32.0%)	0.75
Glomerular disease	11	(25.6%)	3	(16.7%)	8	(32.0%)	0.31
Hereditary/Familial nephropathy	4	(9.3%)	3	(16.7%)	1	(4.0%)	0.29
Others*	13	(30.2%)	5	(27.8%)	8	(32.0%)	1.00
Age at KRT initiation (year), median, (IQR: Q1–Q3)	8.7	(4.5–14.3)	7.7	(3.1–13.1)	9.5	(5.3–14.9)	0.34
Initial KRT modality, *n* (%)							
HD	7	(16.3%)	3	(16.7%)	4	(16.0%)	1.00
PD	35	(81.4%)	15	(83.3%)	20	(80.0%)	1.00
Pre-emptive kidney transplant	1	(2.3%)	0	(0.0%)	1	(4.0%)	1.00
Duration of KRT till Last FU (months), median, (IQR: Q1-Q3)	29.0	(17.0–46.5)	41.0	(29.0–47.0)	25.0	(13.0–35.0)	0.0612
< 28 months, n (%)	19	(44.2	4	(22.2%)	15	(60.0%)	0.0281^
≥ 28 months, n (%)	24	(55.8%)	14	(77.8%)	10	(40.0%)	
History of kidney transplant, n (%)							
Deceased donor	23	(53.5%)	12	(66.7%)	11	(44.0%)	0.22
Living donor	2	(4.7%)	0	(0.0%)	2	(8.0%)	0.50
Nil	18	(41.9%)	6	(33.3%)	12	(48.0%)	0.37
History of immunosuppression use, n (%)							1.00
Yes	31	(72.1%)	13	(72.2%)	18	(72.0%)	
Nil	12	(27.9%)	5	(27.8%)	7	(28.0%)	

^*^Other primary disease includes haemolytic uraemic syndrome, ischaemic nephropathy, Wilms tumour, nephrosclerosis, post-chemotherapy nephrotoxicity, and hepatorenal syndrome

^ Significantly different at α = 0.05

*ACKD* Acquired cystic kidney disease, *CAKUT* Congenital anomalies of the kidney and urinary tract, *FU* follow-up, *IQR* Interquartile range, *KRT* Kidney replacement therapy, *HD* Hemodialysis, *PD* Peritoneal dialysis

### Incidence and clinical presentations of ACKD

ACKD was detected in 18 patients (41.9%), of whom 9 (21%) and 9 (21%) patients had simple and complex cysts, respectively (Fig. 1). Of the 9 subjects with complex ACKD, the cysts were assigned to be grade 2 (*n* = 5), 2F (*n* = 2), and 3 (*n* = 2), respectively (Table 3). Of note, 12 patients (67%) with ACKD were kidney transplant recipients and complex cysts were common (simple ACKD, *n* = 5; complex ACKD, *n* = 7).

**Table 3:** Classification and characteristics of ACKD

Classification of ACKD patients (*N* = 43)		
**No ACKD, n (%)**	**25**	**(58.1%)**
No cyst	8	(32.0%)
Solitary cyst	17	(68.0%)
**ACKD, n (%)**	**18**	**(41.9%)**
Simple cyst (Grade 1)	9	(50.0%)
Complex cyst	9	(50.0%)
Grade 2	5	(27.8%)
Grade 2F	2	(11.1%)
Grade 3	2	(11.1%)

^*^Grading under Bosniak Criteria for ACKD

*ACKD* Acquired cystic kidney disease

The majority of patients with ACKD (89%) were asymptomatic. Two patients (8.7%) had complex cysts (grade 2F, *n* = 1; grade 3, *n* = 1). One patient developed back pain and gross haematuria 72 months after initiation of KRT. Another patient developed cyst infection, complicated by back pain and clinical sepsis 60 months following KRT initiation. One kidney transplant recipient (5.5%) developed recurrent cyst infections and underwent unilateral nephrectomy. He was incidentally diagnosed with ACKD-associated renal cell carcinoma [25].

### Factors associated with the development of ACKD

Compared to those without ACKD, children and adolescents with ACKD had a longer duration of chronic dialysis at the time of analysis (Table 2). By ROC curve analysis, the duration of chronic dialysis ≥ 28 months was the optimal cut-off for the development of ACKD (AUC: 0.669; p < 0.05). A significantly higher proportion of patients with ACKD received long-term dialysis ≥ 28 months (77.8% vs. 40%, *p* = 0.028), compared to those who did not develop ACKD. Other parameters including gender, primary kidney disease, total duration of KRT (including dialysis and kidney transplant), history of kidney transplant, and history of immunosuppression use, were not associated with the detection of ACKD (Table 2). Univariable logistic regression showed that patients with a duration of dialysis of ≥ 28 months had a significantly higher risk of developing ACKD (OR: 6.22, 95% CI: 1.57 to 24.71, *p*-value = 0.009). Multivariate analysis, after adjusting for age, gender, and history of kidney transplant, further confirmed the duration of dialysis of ≥ 28 months as the only predictive factor (OR_adj_: 6.09, CI: 1.43 to 25.82, *p*-value = 0.014). Other factors including gender, age, primary kidney disease, KRT modality, history of kidney transplant, and immunosuppression use were not associated with the development of ACKD (Table 4).

**Table 4:** Univariate and multivariate analysis for various characteristics influencing ACKD

Outcome: ACKD	Univariate analysis ^a^			Multivariate analysis ^b^		
Patient Characteristics (*n* = 43)	Odds ratio	95% CI	*p*-value	Adjusted Odds ratio	95% CI	*p*-value
Gender						
Male	0.689	0.201 to 2.359	0.55	0.700	0.178 to 2.752	0.61
Female	1.000					
Age at 31 Dec 2023 (year)						
< 15	1.273	0.377 to 4.291	0.70	0.943	0.244 to 3.641	0.93
≥ 15	1.000					
Ethnicity						
Chinese	0.435	0.065 to 2.918	0.39			
Non Chinese	1.000					
Primary disease ^c^						
CAKUT	1.000					
Glomerular disease	0.429	0.081 to 2.277	0.32			
Hereditary/Familial nephropathy	3.429	0.287 to 40.948	0.33			
Others*	0.714	0.158 to 3.231	0.66			
Age at KRT initiation (year)						
< 9	2.000	0.583 to 6.867	0.27			
≥ 9	1.000					
Initial KRT modality						
HD	1.000	0.194 to 5.154	1.00			
PD	1.000					
Duration of dialysis till last FU (month)						
≥ 28	6.222	1.567 to 24.709	0.0094	6.085	1.434 to 25.819	0.0143^
< 28	1.000					
History of kidney transplant						
Yes	2.167	0.617 to 7.603	0.23	2.494	0.608 to 10.236	0.205
No	1.000					
History of immunosuppression use						
Yes	1.011	0.262 to 3.905	0.99			
No	1.000					

^a^ Univariable odds ratio presented to assess the association between the independent characteristics and the odds of outcome occurring, without considering the influence of other variables

^b^ The multiple logistic regression model demonstrated statistical significance (Chi-squared value (4) = 8.267, *p* = 0.082), with a Nagelkerke R square of 0.235. The model achieved an accuracy of 0.658, sensitivity of 0.556 and specificity of 0.76 at a cut-off point of 0.5

^c^ Univariable odds ratio presented to assess the association between a single primary disease and other primary diseases

^*^Other primary disease includes haemolytic uraemic syndrome, ischaemic nephropathy, Wilms tumour, nephrosclerosis, post-chemotherapy nephrotoxicity, and hepatorenal syndrome

^ Significantly different at α = 0.05

*ACKD* Acquired cystic kidney disease, *CAKUT* Congenital anomalies of the kidney and urinary tract, *FU* follow-up, *KRT* kidney replacement therapy, *HD* Hemodialysis, *PD* Peritoneal dialysis

We further evaluated the factors that might be associated with the detection of simple and complex cysts among patients with ACKD (Table 5). Older age at KRT initiation (13.2, IQR 8.7–16.0, vs. 2.9, IQR 2.3–6.2, years; *p* = 0.003) and at last follow-up (20.1, IQR 18.2–20.9, vs. 12.1, IQR 9.0–12.6, years; *p* = 0.004) were associated with the detection of complex cysts.

**Table 5:** Subgroup analysis for simple vs. complex cysts

Patient characteristics (*n* = 43)	ACKD (*N* = 18)				*p*-value
	Simple Cysts (*N* = 9)		Complex Cysts (*N* = 9)		
Gender: Male, *n* (%)	3	(33.3%)	4	(44.4%)	1.00
Age at 31 Dec 2023 (year), median, (IQR: Q1–Q3)	12.1	(9.0–12.6)	20.1	(18.2–20.9)	0.004^
Ethnicity, n (%)					1.00
Chinese	8	(88.9%)	7	(77.8%)	
Non Chinese	1	(11.1%)	2	(22.2%)	
Caucasian	0	(0.0%)	1	(11.1%)	
Nepal	0	(0.0%)	0	(0.0%)	
Pakistani	1	(11.1%)	1	(11.1%)	
Primary disease, *n* (%)					
CAKUT	5	(55.6%)	2	(22.2%)	1.00
Glomerular disease	1	(11.1%)	2	(22.2%)	1.00
Hereditary/Familial nephropathy	1	(11.1%)	2	(22.2%)	1.00
Others	2	(22.2%)	3	(33.3%)	1.00
Age at KRT initiation (year), median, (IQR: Q1–Q3)	2.9	(2.3–6.2)	13.2	(8.7–16.0)	0.0031^
Initial KRT modality, n (%)					1.00
HD	2	(22.2%)	1	(11.1%)	
PD	7	(77.8%)	8	(88.9%)	
Pre-emptive kidney transplant	0	(0.0%)	0	(0.0%)	
Duration of dialysis till last FU (months), median, (IQR: Q1–Q3)	46.0	(33.0–47.0)	39.0	(29.0–45.0)	0.66
< 28 months, n (%)	2	(22.2%)	2	(22.2%)	1.00
≥ 28 months, n (%)	7	(77.8%)	7	(77.8%)	
History of kidney transplant, *n* (%)					
Deceased donor	5	(55.6%)	7	(77.8%)	1.00
Living donor	0	(0.0%)	0	(0.0%)	1.00
Nil	4	(44.4%)	2	(22.2%)	0.62
History of immunosuppression use, n (%)					0.29
Yes	5	(55.6%)	8	(88.9%)	
Nil	4	(44.4%)	1	(11.1%)	

Comparison of patients classified as having simple cysts and complex cysts using Fisher exact test for categorical variables and Mann–Whitney U test for continuous variables

^ Significantly different at α = 0.05

*ACKD* Acquired cystic kidney disease, *CAKUT* Congenital anomalies of the kidney and urinary tract, *FU* follow-up, *IQR* Interquartile range, *KRT* Kidney replacement therapy, *HD* Hemodialysis, *PD* Peritoneal dialysis

### Comparison of different imaging modalities

Of the 18 patients with ACKD, 16 patients underwent both ultrasonography and non-contrast MRI for their native kidneys. For the 9 patients with complex cysts (grade 2, *n* = 5; grade 2F, *n* = 2; grade 3, *n* = 2) that were diagnosed by MRI, ultrasonography was able to detect the complex cysts in only 5 patients (grade 2, *n* = 2, grade 2F *n* = 1; grade 3, *n* = 2). For the remaining cases of complex cysts, ultrasonography identified 3 patients with simple cysts, and 1 patient with no cyst. This discrepancy between MRI and ultrasonography may be due to suboptimal visualisation of atrophic native kidneys via ultrasound. In short, MRI was able to detect three additional grade 2 cysts and one additional grade 2F cyst compared to ultrasonography.

Contrast-enhanced CT was performed in 4 patients with high-grade complex cysts (grade 2F, *n* = 2; grade 3, *n* = 2), which were in concordance with the gradings assigned by non-contrast MRI.

## Discussion

Our study shows that ACKD is not uncommon in children and adolescents with kidney failure in the modern era, even among transplant recipients. Importantly, a longer duration of chronic dialysis (≥ 28 months) not only increases the risk of developing ACKD but also could potentially evolve to complex cysts with malignant potential. These findings highlight the importance of early kidney transplant and regular surveillance of ACKD to prevent the development of life-threatening complications.

Compared to previous reports, our study demonstrated a higher incidence of ACKD, where 46% of our paediatric cohort was diagnosed with ACKD. In the historical cohorts, the detection rates of ACKD ranged between 22–90% and 22–46% in adults and children receiving dialysis, respectively [10–14, 20, 21]. The considerable variations in the reported incidence of ACKD are largely due to heterogeneous study designs, imaging strategies, treatment era, as well as patient populations that might experience discrepant waiting time to kidney transplant depending on the local transplant policies. Another plausible explanation for the higher observed disease incidence is an improvement in imaging techniques and qualities, leading to a higher and more reliable detection of ACKD. Furthermore, the detection of ACKD in our study was enhanced by utilizing both ultrasonography and MRI. There are limited reports on ACKD in the recent decade, where the incidence of ACKD was reported to be 20 to 31.6% in adults [26, 27], and 35% in children on maintenance haemodialysis [28].

Our results demonstrate that a duration of dialysis ≥ 28 months increased the risk of developing ACKD by sixfold, when compared to a shorter duration of dialysis. This is in concordance with published data from the adult and paediatric populations [14, 20, 29]. In adults, 10–20% and 40–60% of patients develop ACKD by 3 and 5 years of dialysis, respectively [14]. By 10 years of dialysis, more than 90% of patients develop ACKD [14, 20, 29]. Previous paediatric data from Matteo et al*.* showed that the time from dialysis initiation to first detection of ACKD was 50.3 ± 15.2 months [13]. Similar findings were also observed in children on haemodialysis, where a significantly higher proportion of patients developed ACKD after receiving dialysis for > 48 months [28]. In contrast, the total duration of KRT (including dialysis and transplant) was not associated with the development of ACKD. This further affirms the role of chronic dialysis in the disease pathogenesis and stresses the importance of early transplantation. In Hong Kong, the reported median waiting time to kidney transplant for paediatric patients was 3.7 years [6]. This is considerably longer than other Western countries: United States (197 days), United Kingdom (351 days), and Europe (1.8 years) [30–32]. A major challenge for early kidney transplant is a low kidney donation rate in our locality (6.3 pmp) [33]. This leads to long waiting time to transplant, which is particularly relevant to infants receiving chronic dialysis [7]. This may also delay the presentation of ACKD and associated complications after transferring to adult medical care [34]. It is hoped that with further promotion of organ donation and living-related transplantation, prioritization of organ allocation to paediatric patients, and the development of specific transplant initiatives such as paired kidney donation, can help to expand the donor pool and alleviate the situation.

While our findings demonstrate that 89% of ACKD patients were asymptomatic, all significant complications occurred with complex cysts, including severe infection and renal cell carcinoma. Although only one patient developed ACKD-associated renal cell carcinoma in our cohort, there were significant treatment implications on his transplant care, including the use of immunosuppression and the risk of potential rejection. Furthermore, 58% of our patients with ACKD were kidney transplant recipients. This is contrary to previous reports where lower cyst grades and even regression of ACKD were observed after kidney transplant [35, 36]. One potential explanation is related to uremic serum which exerts a growth-promoting effect on kidney cells in vitro [37]. Following successful kidney transplant, levels of growth factors decrease due to lower serum urea levels. However, renal cell carcinoma has been reported after kidney transplant [12, 20]. Thus, regular screening for ACKD should be performed in children receiving all forms of KRT, including kidney transplant, for early ACKD and timely intervention to prevent complications. Ultrasonography is conventionally considered as the preferred imaging modality for ACKD surveillance because it is non-invasive, non-radiating, relatively economical and readily available. In our study, we attempted to compare the detection rates of ACKD, and the ability to differentiate simple and complex cysts between ultrasonography and non-contrast MRI. Gadolinium-based contrast agent was not used in MRI due to the potential risk of developing nephrogenic systemic fibrosis in patients with kidney failure. It was noted that more than one-third of our patients with complex cysts were missed by ultrasonography, compared to non-contrast MRI. Ultrasonography has limited sensitivity in detecting small cysts, and its performance is operator-dependent. In a prospective study comprising 201 patients, ultrasonography identified 26%, 60%, and over 80% of CT-confirmed kidney lesions that measured < 1 cm, 1–2 cm, and > 2 cm, respectively [38]. Non-contrast MRI may therefore be preferred in patients with known ACKD to better characterize the cyst nature. However, MRI is often less readily available than ultrasonography in daily clinical practice. Therefore, MRI may be considered in selected patients with a higher risk of developing complex cysts. Our data indicate that these patients are those on long-term chronic dialysis, or older age at KRT initiation and last follow-up. The Bosniak criteria for cyst evaluation was originally designed and validated for contrast-enhanced CT, although it has been extrapolated to MRI studies [39]. In our study, contrast-enhanced CT was performed in cysts that are suspected to be Bosniak II or above, and there was good concordance for cyst gradings between plain MRI and contrast-enhanced CT examinations. Reliable assignment of cyst grading is imperative, as high-grade complex cyst predicts malignant potential and requires timely intervention such as nephrectomy.

To date, there is no local or international consensus pertaining to the surveillance of ACKD in children and adolescents with kidney failure. For paediatric patients, a screening algorithm comprising annual ultrasonography on both native and graft kidneys was previously proposed by our group [15]. If no cysts or only simple cysts are detected, annual ultrasonography should be continued. Non-contrast MRI should be performed if complex cysts are detected on ultrasonography. Our current findings support this recommendation. In addition, we suggest that non-contrast MRI may be preferred in those receiving dialysis > 3 years owing to an increased risk of developing complex cysts, which may be missed by ultrasonography. For high-grade complex cysts (Bosniak grade 2 or above), contrast-enhanced CT is recommended for proper classification. Patients with cysts of grade 3 or 4 should be timely referred to a paediatric urologist for nephrectomy.

The strength of our study is the territory-wide patient representation with minimal missing data. However, there are several limitations. First, this is a cross-sectional study. Regular radiological surveillance was not previously performed, thus reliable determination of the disease onset of ACKD is not feasible. Second, our study has a small sample size which limits the feasibility of further subgroup analyses. In particular, patients with CAKUT may be associated with cystic changes and cystic kidney disease, such as nephronophthisis, may develop cysts over time [40]. We therefore excluded these patients in the current study for the purpose of research. The reason for this exclusion was that it was not radiologically feasible to determine whether the detected cysts were congenital or hereditary, or acquired during the progression of chronic kidney disease. This is particularly true as previous imaging studies were often performed by ultrasonography, which is operator dependent. Therefore, only CAKUT patients without cystic changes prior to the development of kidney failure were included. In clinical practice, we believe screening of cysts should be performed in these patients, and probably a more sophisticated and objective imaging technique such as MRI should be employed for better cyst characterization.

Thirdly, CT was only performed selectively in our patients to limit radiation exposure. Gadolinium-based contrast agent was also not used in MRI studies due to the risk of developing nephrogenic systemic fibrosis [41]. These imaging arrangements of the cysts may confound our results. Finally, the data validating the use of the Bosniak criteria to estimate malignancy risk are limited to the adult population.

Also, while all eligible patients had participated in the current study, 28 subjects were excluded from the recruitment since they were no longer receiving our active care, nephrectomised, or having an underlying kidney condition that might account for the cystic changes. Further investigations are required to evaluate the rates of ACKD in these specific populations. Lastly, the study is limited by its retrospective nature and is subject to selection and reporting bias.

In conclusion, our study shows that ACKD is common in children and adolescents with kidney failure, even among kidney transplant recipients. Longer duration of dialysis is the only significant factor predictive of ACKD. While most patients are asymptomatic, serious complications may arise. Importantly, complex cysts may not be identified by ultrasonography alone, thus additional MRI should be considered in selected patients, such as for those undergoing long duration of dialysis. Hence, regular surveillance for ACKD, with ultrasonography and non-contrast MRI is recommended. Future prospective longitudinal studies in this specific patient population are necessary to understand the natural course of ACKD during dialysis and after kidney transplantation. Moreover, it is possible that ACKD can develop over time with CKD progression. Thus, further studies are needed to evaluate the relationship between CKD progression and the development of ACKD.

## Supplementary information

Below is the link to the electronic supplementary material.

Graphical abstract (PPTX 89 KB)

Supplementary file2 (DOCX 26 KB)

## Data Availability

Researchers interested in using more detailed patient-level data could obtain them upon request to the corresponding authors.
