# Supplementary material for: Acquired cystic kidney disease in children with kidney failure
Source: Pediatr Nephrol. 2025 Jan 4;40(5):1741–50. doi: 10.1007/s00467-024-06628-7 (PMC11947055; doi:10.1007/s00467-024-06628-7)
Supplement: Supplementary file 1 — Graphical abstract (PPTX 89 KB) [file 467_2024_6628_MOESM1_ESM.pptx]

## Slide 1
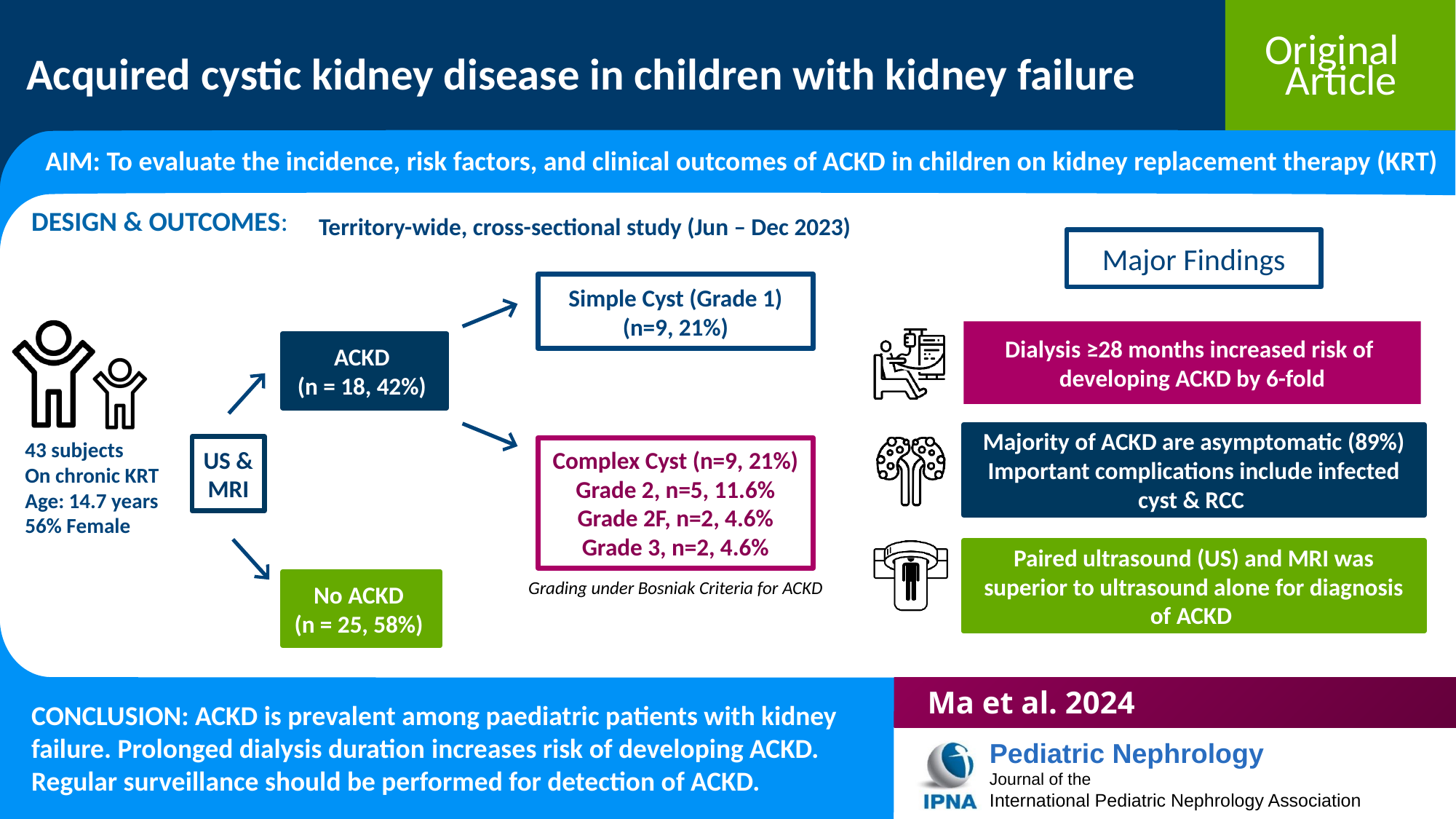

Acquired cystic kidney disease in children with kidney failure
AIM: To evaluate the incidence, risk factors, and clinical outcomes of ACKD in children on kidney replacement therapy (KRT)
DESIGN & OUTCOMES:
Territory-wide, cross-sectional study (Jun – Dec 2023)
Major Findings
Simple Cyst (Grade 1)
(n=9, 21%)
Dialysis ≥28 months increased risk of
developing ACKD by 6-fold
ACKD
(n = 18, 42%)
Majority of ACKD are asymptomatic (89%)
Important complications include infected cyst & RCC
43 subjects
On chronic KRT
Age: 14.7 years
56% Female
US & MRI
Complex Cyst (n=9, 21%)
Grade 2, n=5, 11.6%
Grade 2F, n=2, 4.6%
Grade 3, n=2, 4.6%
Paired ultrasound (US) and MRI was superior to ultrasound alone for diagnosis of ACKD
Grading under Bosniak Criteria for ACKD
No ACKD
(n = 25, 58%)
Ma et al. 2024
CONCLUSION: ACKD is prevalent among paediatric patients with kidney failure. Prolonged dialysis duration increases risk of developing ACKD. Regular surveillance should be performed for detection of ACKD.
