# Supplementary material for: Acquired cystic kidney disease in children with kidney failure
Source: Pediatr Nephrol. 2025 Jan 4;40(5):1741–50. doi: 10.1007/s00467-024-06628-7 (PMC11947055; doi:10.1007/s00467-024-06628-7)
Supplement: Supplementary file 2 — Supplementary file2 (DOCX 26 KB) [file 467_2024_6628_MOESM2_ESM.docx]

**Supplementary Figure 1:**

**Receiver operating characteristic (ROC) for the duration of dialysis in ACKD patients**


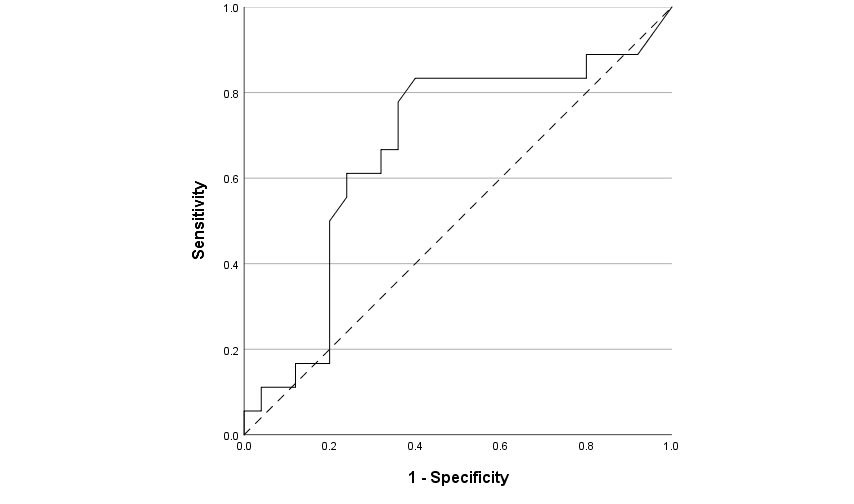


**AUC=0.669; 95%CI: 0.496 - 0.842**

Remarks:
1) Area Under the ROC Curve is 0.669, 95%C.I.: 0.496-0.842
2) Optimal cut-off of duration of dialysis in the classification of ACKD patients >= 28 months;

Sensitivity = 83.3%; Specificity = 60.0%
